# PINK1/TAX1BP1-directed mitophagy attenuates vascular endothelial injury induced by copper oxide nanoparticles

**DOI:** 10.1186/s12951-022-01338-4

**Published:** 2022-03-19

**Authors:** Yinzhen Fan, Zhenli Cheng, Lejiao Mao, Ge Xu, Na Li, Mengling Zhang, Ping Weng, Lijun Zheng, Xiaomei Dong, Siyao Hu, Bin Wang, Xia Qin, Xuejun Jiang, Chengzhi Chen, Jun Zhang, Zhen Zou

**Affiliations:** 1 Molecular Biology Laboratory of Respiratory Disease, Institute of Life Sciences, Chongqing Medical University, Chongqing, 400016 People’s Republic of China; 2 Department of Cardiovascular Medicine, Children’s Hospital of Chongqing Medical University, Chongqing, 400014 People’s Republic of China; 3 Chongqing Key Laboratory of Pediatrics, Children’s Hospital of Chongqing Medical University, Chongqing, 400014 People’s Republic of China; 4 College of Pharmacy, Chongqing Medical University, Chongqing, 400016 China; 5 Department of Pharmacy, The First Affiliated Hospital of Chongqing Medical University, Chongqing, 400016 China; 6 Center of Experimental Teaching for Public Health, Experimental Teaching and Management Center, Chongqing Medical University, Chongqing, 400016 People’s Republic of China; 7 Department of Occupational and Environmental Health, School of Public Health and Management, Chongqing Medical University, Chongqing, 400016 China; 8 Dongsheng Lung‒Brain Diseases Joint Laboratory, Chongqing Medical University, Chongqing, 400016 China

**Keywords:** CuONPs, Vascular endothelial injury, Mitophagy, PINK1/TAX1BP1, Urolithin A

## Abstract

Copper oxide nanoparticles (CuONPs) are widely used metal oxide NPs owing to their excellent physical–chemical properties. Circulation translocation of CuONPs after inhalation leads to vascular endothelial injury. Mitochondria, an important regulatory hub for maintaining cell functions, are signaling organelles in responses to NPs-induced injury. However, how mitochondrial dynamics (fission and fusion) and mitophagy (an autophagy process to degrade damaged mitochondria) are elaborately orchestrated to maintain mitochondrial homeostasis in CuONPs-induced vascular endothelial injury is still unclear. In this study, we demonstrated that CuONPs exposure disturbed mitochondrial dynamics through oxidative stress-dependent manner in vascular endothelial cells, as evidenced by the increase of mitochondrial fission and the accumulation of fragmented mitochondria. Inhibition of mitochondrial fission with Mdivi-1 aggravated CuONPs-induced mtROS production and cell death. Furthermore, we found that mitochondrial fission led to the activation of PINK1-mediated mitophagy, and pharmacological inhibition with wortmannin, chloroquine or genetical inhibition with siRNA-mediated knockdown of PINK1 profoundly repressed mitophagy, suggesting that the protective role of mitochondrial fission and PINK1-mediated mitophagy in CuONPs-induced toxicity. Intriguingly, we identified that TAX1BP1 was the primary receptor to link the ubiquitinated mitochondria with autophagosomes, since *TAX1BP1* knockdown elevated mtROS production, decreased mitochondrial clearance and aggravated CuONPs-induced cells death. More importantly, we verified that urolithin A, a mitophagy activator, promoted mtROS clearance and the removal of damaged mitochondria induced by CuONPs exposure both in vitro and in vivo. Overall, our findings indicated that modulating mitophagy may be a therapeutic strategy for pathological vascular endothelial injury caused by NPs exposure.

**Graphical Abstract:**

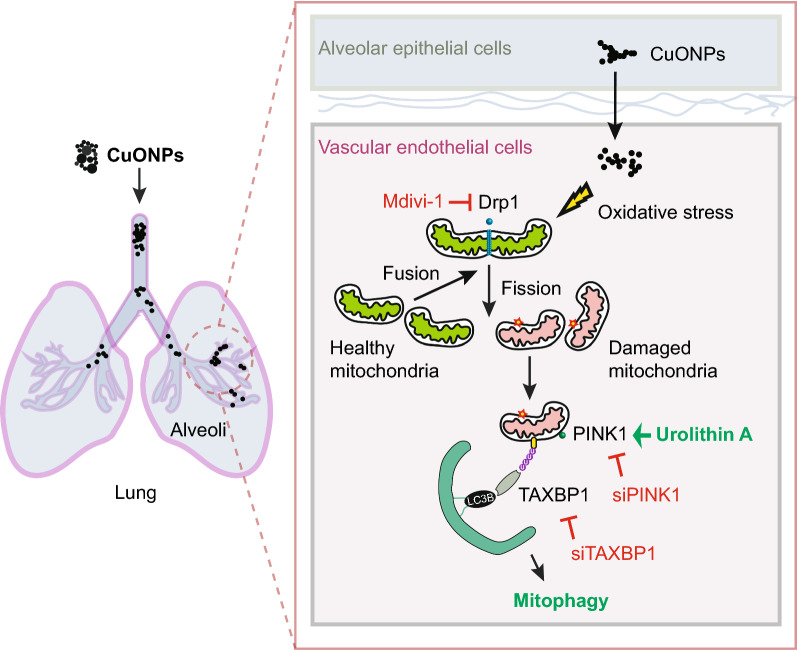

## Highlights

1. CuONPs disturb mitochondrial dynamics and trigger mitophagy in vascular endothelial cells and mouse blood vessel.
2. PINK1/TAX1BP1-mediated mitophagy regulates the removal of excessive ROS and aberrant mitochondria in CuONPs-treated vascular endothelial cells.
3. The mitophagy activator urolithin A attenuates CuONPs-induced vascular endothelial cells death and mice vascular injury.

## Introduction

Copper oxide nanoparticles (CuONPs) are widely used in industrial and biomedical fields owing to their excellent physical–chemical properties, earth-abundant and inexpensive properties [1]. For example, CuONPs have been efficiently used for biological sensors to detect glucose, cholesterol, disease-related protein biomarkers and others [2]. CuONPs nanomedicines have been used against various types of tumors [3–5]. In addition, CuONPs show excellent antimicrobial activities against *Staphylococcus aureus* and *Escherichia coli* [6]. Currently, CuONPs is also developed as antiviral surface coatings suppressing the spread of SARS-CoV-2 [7–9]. Meanwhile, CuO nanocomposites are designed as a potential treatment against bacterial infected diabetic non-healing wounds [10, 11].

However, the widespread application of CuONPs in industrial and biomedical fields has seriously threatened the health of this human being. CuONPs are recognized as particularly highly toxic NPs, when compared to many other metal oxide NPs [1, 12]. Accumulating evidence shows that the transformation of nanomaterials in the environment or living systems is closely related to nanomaterials stability and toxicity [13, 14]. CuONPs also undergo chemical transformation under conditions relevant to living systems and the natural environment. CuONPs dissolve at lysosomal pH (4–5) solution and undergo sulfidation by a dissolution-reprecipitation mechanism [15]. Consistently, in our previous in-vitro study, we confirmed that lysosomal deposition of CuONPs facilitated the release of Cu ions from CuONPs-treated vascular endothelial cells [16]. However, the underlying protective mechanisms against CuONPs toxicity are not yet fully understood.

Inhaled NPs are closely linked to cardiovascular diseases [17–19]. Exposure to combustion-derived NPs impairs vascular physiological functions and inhibits arterial vasodilatation both in pre-clinical (rat model) and clinical model [20]. Pulmonary exposure to engineered NPs results in oxidative stress and inflammation, consequently contributes to the progression of atherosclerosis [21–23]. Vascular endothelial cells cover the inner surface of all blood vessels and serve as a regulatory hub of systemic circulation [24]. Because inhaled NPs can translocate into systemic circulation and accumulate at the sites of vascular disease, NPs may directly interact with vascular endothelial cells and impair vascular physiological functions [25].

Mitochondrial dynamics is an important metabolic and regulatory hub characterized by mitochondrial fusion, fission and degradation of damaged mitochondria [26]. Disruption of mitochondrial dynamics leads to multiple diseases such as cancers, cardiovascular disease, neurodegenerative disease, aging and others [27]. We previously reported that CuONPs treatment resulted in mitochondrial dysfunction and excessive mitochondrial ROS (mtROS) production in vascular endothelial cells [16, 28, 29]. Nonetheless, whether the mitochondrial dynamics is involved in in CuONPs-induced vascular endothelial injury is still unclear.

Mitophagy is a selective autophagic process that specifically degrading damaged mitochondria by autophagy-lysosome machinery to maintain mitochondrial homeostasis [30, 31]. Mitophagy is regulated by the ubiquitin kinase PTEN induced kinase 1 (PINK1) and mitophagy receptors. Several studies have demonstrated mitophagy is involved in nanoparticle-induced cytotoxicity including human fetal hepatocyte line L-02 [32], human hepatocellular carcinoma (HepG2) cells [33, 34], human lung adenocarcinoma (A549) cells [35] and murine microglia cell line (BV-2) [36]. In particular, we previously also reported that mitophagy was activated in CuONPs-treated human umbilical vein endothelial cells (HUVECs) [29]. However, the mechanisms of mitophagy in CuONPs-induced vascular endothelial injury is still not fully understood.

In the current study, we demonstrated the involvement of mitochondrial dynamics and mitophagy in CuONPs exposure-induced vascular endothelial injury in vitro and in vivo. In addition, we investigated the roles of urolithin A (UA), which is a first-in-class natural compound that inducing mitophagy. This study uncovers the importance of mitophagy-medicated mitochondrial homeostasis in CuONPs-medicated cytotoxicity and highlights the potential application of mitophagy activator in preventing NPs-triggered vascular injury.

## Results

### CuONPs disturbs mitochondrial dynamics in vascular endothelial cells

**Fig. 1:**
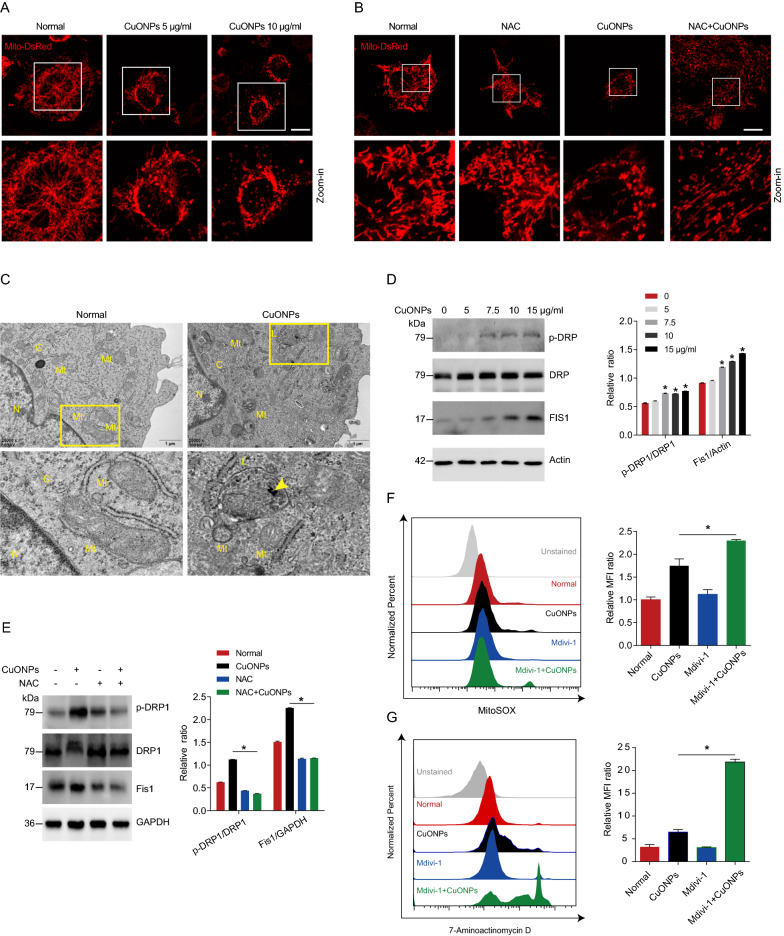
CuONPs induced disturbance of mitochondrial dynamics. **A** Representative fluorescence images of the stable EA.hy926 cell lines expressing Mito-DsRed after treatment with low or high dose CuONPs (5 and 10 μg/ml, respectively) for 12 h. Scale bars, 20 μm. **B** Representative fluorescence images of Mito-DsRed cells which were pretreated with NAC (10 mM) and then treated with CuONPs (10 μg/ml). Scale bars, 20 μm. **C** Representative TEM images of EA.hy926 cells after treatment with CuONPs for 12 h. N, nucleus; C, cytoplasm; Mt, mitochondria; L, lysosomes. Yellow arrow indicates uptaked CuONPs. **D** Western blotting analysis and quantification of the protein levels of p-DRP1, DRP and Fis1 in EA.hy926 treated with 0, 5, 7.5, 10 and 15 μg/ml CuONPs for 12 h, respectively. Actin was served as internal control. **E** Western blotting analysis and quantification of the protein levels of p-DRP1, DRP and Fis1 in EA.hy926 treated with NAC (10 mM) and CuONPs (10 μg/ml). GAPDH was used as internal control. **F** Representative FCM results of EA.hy926 cells after MitoSOX staining. The cells were pretreated with or without Mdivi-1 (25 μM) for 1 h, and then were treated with CuONPs (10 μg/ml) for 12 h. MFI, mean fluorescence intensity. **G** Representative FCM results of EA.hy926 cells after 7-Aminoactinomycin D (7-AAD) staining. In **D**, two-tailed unpaired Student’s *t* test was performed for statistical analysis. In **E**, **F** and **G**, one-way ANOVA with Tukey’s test was used for multiple comparisons. Results are representative of at least three independent experiments. Data are mean ± S.D. *p < 0.05

Physical properties of CuONPs have been reported in our previous study [16, 37]. Briefly, transmission electron microscopy (TEM) analysis showed that CuONPs were spherical NPs with a diameter size of about 50 nm. The spectra of field emission scanning electron microscope/energy dispersive X-ray spectroscopy (FE/SEM–EDS) showed strong peaks of Cu and O specific peaks. Dynamic light scattering (DLS) analysis showed that the average of hydrodynamic radius and zeta potential in the culture medium were 183.70 nm and − 13.17 mV. To investigate whether CuONPs undermine mitochondrial dynamics, we constructed a EA.hy926 vascular endothelial cell line stable expressing Mito-DsRed, which labels mitochondria with DsRed fluorescent signal in live cells (Fig. 1A). Mito-DsRed cells were treated with low- or high-dose of CuONPs (5 μg/ml or 10 μg/ml, respectively) for 12 h and detected under confocal microscope. Live cell imaging showed that CuONPs induced abnormal changes in mitochondrial morphology characterized by accumulation of fragmented (short tubes) mitochondria in CuONPs-treated cells, indicating that CuONPs treatment promotes mitochondrial fission in vascular endothelial cells (Fig. 1A). Moreover, we showed that N-Acetyl-L-cysteine (NAC), a scavenger of ROS, obviously prevented CuONPs-induced mitochondrial fission, suggesting ROS is an up-stream regulator regulating mitochondrial fission in CuONPs-treated vascular endothelial cells (Fig. 1B). Additionally, TEM images showed that CuONPs indeed damaged mitochondrial dynamics characterized by accumulation of fragmented mitochondria in cells (Fig. 1C). Mitochondrial fission is regulated by a dynamin superfamily of GTPases dynamin related protein 1 (Drp1) and fission protein 1 (Fis1) [38], therefore we analyzed changes in phospho-Drp1 (Drp1 activation form) and Fis1 levels in CuONPs-treated cells via western blotting. The results showed that p-Drp1 (Ser616) and Fis1 were upregulated in CuONPs-treated cells, suggesting that CuONPs induce mitochondrial fission in vascular endothelial cells (Fig. 1D). Moreover, we showed that NAC obviously prevented upregulation of p-Drp1 (Ser616) and Fis1 induced by CuONPs in EA.hy926 cells (Fig. 1E). We then investigated the roles of mitochondrial fission in CuONPs-induced cytotoxicity. Flow cytometry (FCM) results showed that Mdivi-1, a mitochondrial fission inhibitor, significantly exacerbated CuONPs-induced mtROS production in vascular endothelial cells (Fig. 1F). Importantly, Mdivi-1 aggravated CuONPs-induced cell death in EA.hy926 cells (Fig. 1G). Taken together, these results reveal that CuONPs cause mitochondrial dysfunctions and induce mitochondrial fission. Furthermore, mitochondrial fission exerts protective roles in CuONPs-treated vascular endothelial cells.

### Mitophagy contributes to clearance fragmented mitochondria in CuONPs-treated vascular endothelial cells

**Fig. 2:**
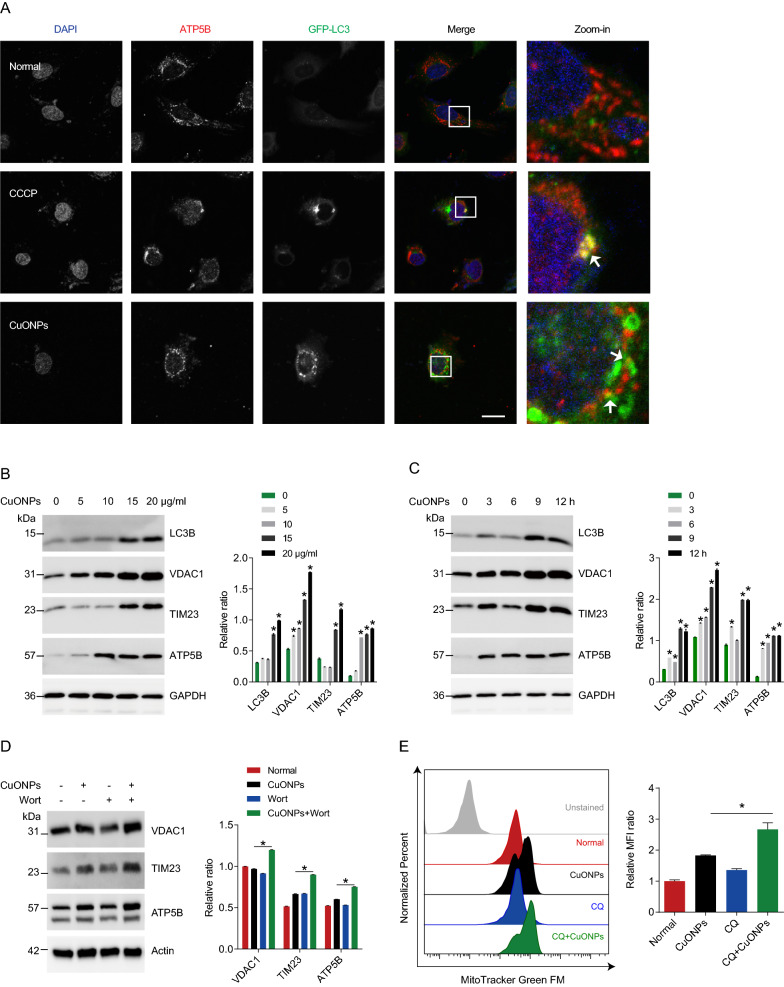
Mitophagy facilitates removal of damaged mitochondria in CuONPs-treated cells. **A** Representative immunofluorescence images of colocalization of GFP-LC3 and ATP5B in EA.hy926 cells treated with CuONPs (10 μg/ml). White arrows indicate colocalized dots of mitochondrial marker ATP5B with autophagosome marker GFP-LC3. Scale bars, 20 μm. **B** Western blotting analysis and quantification of protein level of LC3B, VDAC1, TIM23 and ATP5B in EA.hy926 treated with CuONPs (0, 5, 10, 15 and 20 μg/ml, respectively) for 12 h. **C** Western blotting analysis and quantification of protein level of LC3B, VDAC1, TIM23 and ATP5B in EA.hy926 treated with 10 μg/ml CuONPs for 0, 3, 6, 9 or 12 h. In (B) and (C), two-tailed unpaired Student’s *t* test was performed for statistical analysis. GAPDH was used as internal control. **D** The protein expression levels of VDAC1, TIM23 and ATP5B in EA.hy926 treated with or without wortmannin (Wort). Actin was used as internal control. **E** Representative FCM results of EA.hy926 cells labeled with MitoTracker Green FM. The cells were pretreated with or without chloroquine (CQ) for 1 h and then treated 10 μg/ml CuONPs for 12 h. MFI, mean fluorescence intensity. In **D** and **E**, one-way ANOVA with Tukey’s test was used for statistical analysis. All data are representative of at least three independent experiments. Data are mean ± S.D. *p < 0.05

Mitophagy is a finely regulated process that maintains cellular homeostasis through clearing dysfunctional mitochondria and excessive mtROS in cells [39]. Previous studies have showed that mitochondrial fission acts as a trigger of mitophagy process [40–42]. To determine whether mitophagy is triggered in CuONPs-treated EA.hy926 cells, we constructed a GFP-LC3 stable cell line which labels autophagosome with green fluorescent probe. Fluorescence images showed that CuONPs treatment led to more co-localization of GFP-LC3 and mitochondrial protein ATP5B (Fig. 2A), suggesting mitophagy was activated. Interestingly, both autophagy marker LC3B and mitochondria related markers (VDAC1, TIM23 and ATP5B) were upregulated by CuONPs, indicating that CuONPs induce fragmented mitochondria accumulation in vascular endothelial cells (Fig. 2B, C). To determine whether mitophagy mediates clearance of fragmented mitochondria, we pretreated EA.hy926 cells with autophagy inhibitor wortmannin and found it significantly increased protein levels of mitochondria related markers VDAC1, TIM23 and ATP5B (Fig. 2D). FCM results showed that another autophagy inhibitor chloroquine caused more fragmented mitochondria accumulation in CuONPs-treated EA.hy926 cells (Fig. 2E). Thus, mitophagy facilitates removal of damaged mitochondria in CuONPs-treated vascular endothelial cells.

### CuONPs triggers PINK1-dependent mitophagy in vascular endothelial cells

**Fig. 3:**
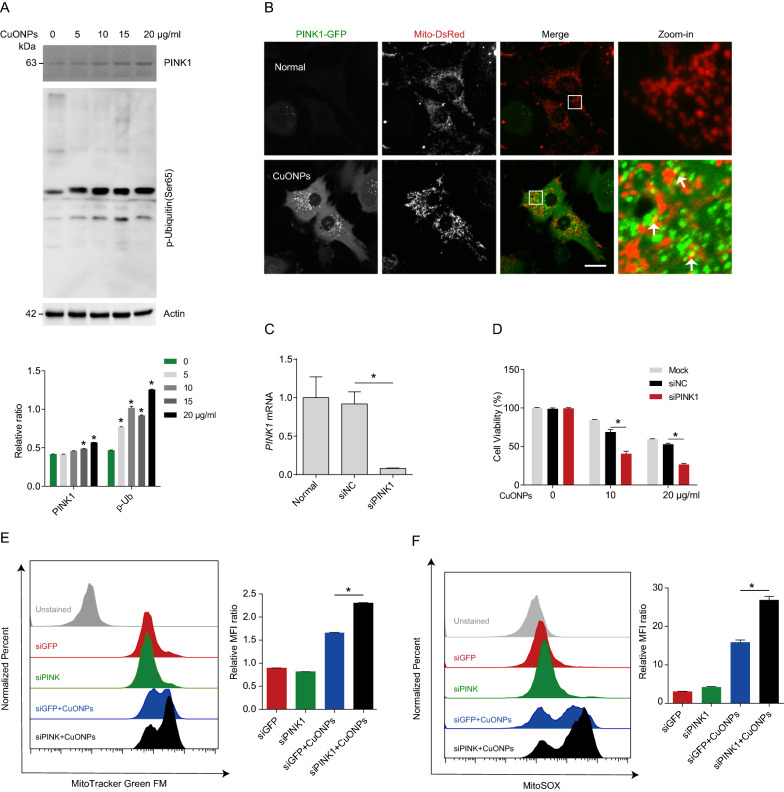
The ubiquitin kinase PINK1 was activated in CuONPs-treated cells. **A** Western blotting analysis and quantification of the protein levels of PINK1 and p-ubiquitin (Ser65) in EA.hy926 cells treated with CuONPs (0, 5, 10, 15 and 20 μg/ml) for 12 h. Actin was used as internal control. **B** Representative fluorescence images of colocalization of PINK1-GFP with Mito-DsRed in EA.hy926 treated with CuONPs (10 μg/ml). White arrows indicate PINK1-positive dots that colocalized with Mito-DsRed signals. Scale bars, 20 μm. **C** RT-qPCR analysis was performed to verify the knockdown efficiency of *PINK1* siRNA. **D** MTS analysis was performed to detect EA.hy926 cell viability. The cells were transfected with siNC or siPINK1 for 48 h and then treated with CuONPs (10 μg/ml) for 12 h. **E** and **F** Representative FCM results of EA.hy926 cells labeled with MitoTracker Green FM (**E**) and MitoSOX (**F**). MFI, mean fluorescence intensity. In **A** and **C**, two-tailed unpaired Student’s *t* test was performed for statistical analysis. In **D**, **E** and **F**, one-way ANOVA with Tukey’s test was used for multiple comparisons. Results are representative of at least three independent experiments. Data are mean ± S.D. *p < 0.05

The ubiquitin kinase PTEN-induced kinase 1 (PINK1) is shown to recruit autophagy receptors to trigger mitophagy [31, 43]. Next, we assessed whether PINK1 mediates the activation of mitophagy and clearance of damaged mitochondria in CuONPs-treated EA.hy926 cells. Western blotting results demonstrated that CuONPs increased PINK1 protein level and the phosphorylation of ubiquitin (a downstream target of PINK1 kinase) in EA.hy926 cells (Fig. 3A). Then, we constructed a EA.hy926 cell line stable co-expressing PINK1-GFP and Mito-DsRed. The results showed that CuONPs recruited PINK1-GFP to Mito-DsRed (labeling mitochondria) (Fig. 3B), suggesting that CuONPs trigger PINK1-dependent mitophagy. To determine the roles of PINK1 in CuONPs-induced cytotoxicity, we knocked down *PINK1* expression in EA.hy926 cells using specific small interfering RNAs (siRNAs) (Fig. 3C). FCM results showed that *PINK1* knockdown increased CuONPs-induced accumulation of damaged mitochondria and mtROS in EA.hy926 (Fig. 3E, F). Furthermore, we found that *PINK1* knockdown exacerbated CuONPs-induced cell death in EA.hy926 (Fig. 3D). Our data thus support that PINK1-dependent mitophagy facilitates clearance of damaged mitochondria in CuONPs-treated EA.hy926 and protects against CuONPs-induced cell death in EA.hy926.

### TAX1BP1 serves as a major mitophagy receptor in CuONPs-treated vascular endothelial cells

**Fig. 4:**
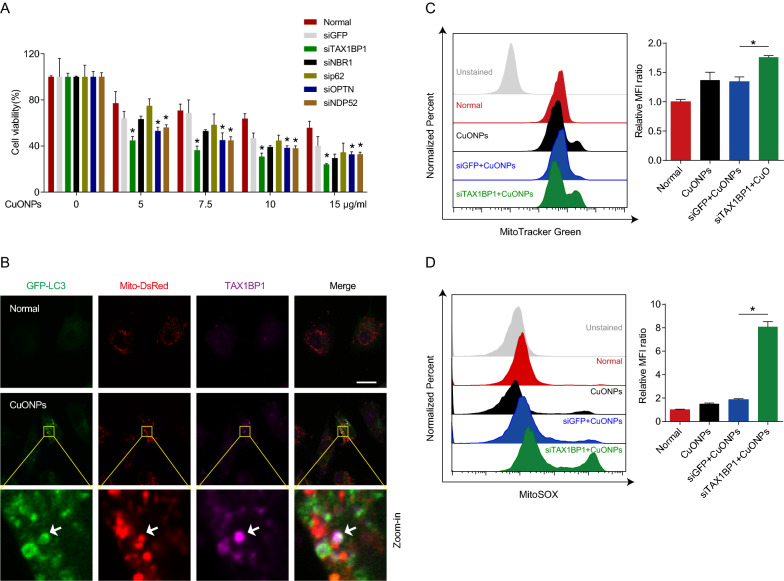
TAX1BP1 was the major receptor of CuONPs-induced mitophagy in vascular endothelial cells. **A** MTS analysis was performed to detect EA.hy926 cell viability. Cells were transfected with siNC, siGFP, siTAX1BP1, siNBR1, sip62, siOPTN or siNDP52 for 48 h, and then treated with CuONPs (0, 5, 7.5, 10 and 15 μg/ml) for 24 h. **B** Representative immunofluorescence images indicates colocalization of TAX1BP1 with GFP-LC3 and Mito-DsRed dots in EA.hy926 stable cell line expressing GFP-LC3 and Mito-DsRed after treatment with 10 μg/ml CuONPs for 12 h. White arrow indicate TAX1BP1-positive dots that colocalized with GFP-LC3 and Mito-DsRed signals. Scale bars, 20 μm. **C** and **D** Representative FCM results of EA.hy926 cells labeled with MitoTracker Green FM (**C**) and MitoSOX (**D**), respectively. The cells were transfected with siNC or siTAX1BP1 for 48 h and then treated with CuONPs (10 μg/ml) for 12 h. All data were statistically analyzed using one-way ANOVA with Tukey’s test. Results are representative of at least three independent experiments. Data are mean ± S.D. *p < 0.05

Because mitophagy requires autophagy receptors to linking ubiquitinated mitochondria with autophagosomes [44, 45]. To determine which receptor mediates removal of damaged mitochondria in CuONPs-treated vascular endothelial cells, we designed siRNAs specific targeting receptor genes including *TAX1BP1*, *NBR1*, *p62*, *OPTN* and *NDP52* respectively. Cell viability results showed that *TAX1BP1* knockdown caused more cell death than other receptor genes in CuONPs-treated EA.hy926 cells, suggesting that TAX1BP1 is the primary receptor mediating removal of damaged mitochondria induced by CuONPs (Fig. 4A). Then, we labeled mitochondria and autophagosomes using Mito-DsRed and GFP-LC3, respectively. Immunofluorescence assay showed that CuONPs treatment induced co-localization of TAX1BP1 with Mito-DsRed and GFP-LC3, indicating that CuONPs recruited TAX1BP1 to mitophagosomes in EA.hy926 cells (Fig. 4B). To further determine the function of TAX1BP1 during mitophagy, we detected mitochondria mass and mtROS level in *TAX1BP1* knockdown cells. FCM results showed that *TAX1BP1* knockdown inhibited mitophagy-mediated removal of damaged mitochondria, resulting in more mitochondria accumulation in CuONPs-treated EA.hy926 cells (Fig. 4D). Moreover, *TAX1BP1* knockdown induced more mtROS accumulation in CuONPs-treated EA.hy926 cells (Fig. 4C). This suggests TAX1BP1 is necessary for CuONPs-mediated mitophagy in vascular endothelial cells.

### Urolithin A induces PINK1-dependent mitophagy and rescues CuONPs-induced vascular endothelial cell death

**Fig. 5:**
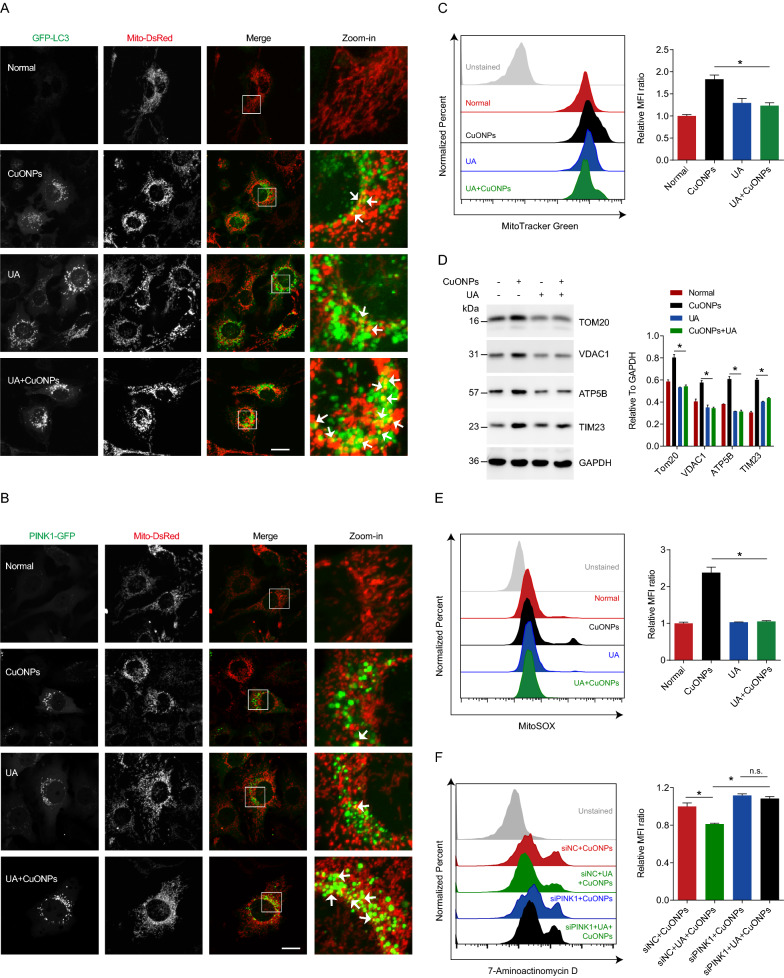
UA alleviated CuONPs-induced cell death. **A** Representative fluorescence images of EA.hy926 cell line stable expressing GFP-LC3 and Mito-DsRed. **B** Representative fluorescence images of EA.hy926 cell line stable expressing PINK1-GFP and Mito-DsRed. In **A** and **B**, the cells were treated with UA (100 μM), CuONPs (10 μg/ml) and UA + CuONPs for 12 h, respectively. White arrows indicate PINK1-positive or LC3-positive dots that colocalized with damaged mitochondria signals. Scale bars, 20 μm. **C** Representative FCM results of EA.hy926 staining with mitochondrial mass marker MitoTracker Green FM. MFI, mean fluorescence intensity. **D** Western blotting analysis and quantification of protein levels of TOM20, VDAC1, ATP5B and TIM23 in CuONPs-treated EA.hy926 cells with or without UA. **E** Representative FCM results of EA.hy926 staining with mtROS probe MitoSOX. **F** Representative FCM results of EA.hy926 staining with 7-AAD. EA.hy926 cells were transfected with siNC or siPINK1 for 48 h, and then treated with UA (100 μM) and CuONPs (10 μg/ml) for 24 h. n.s., not significance. The cells were treated with CuONPs (10 μg/ml) or UA + CuONPs for 12 h, respectively. All data were statistically analyzed using One-way ANOVA with Tukey’s test. The results are representative of at least three independent experiments. Data are mean ± S.D. *p < 0.05

Urolithin A (UA) is a gut microbiome-derived natural product derived from ingested ellagitannins and ellagic acid by gut bacteria [46]. Previous studies have shown that UA induces mitophagy and alleviates a variety of diseases such as aging-related diseases [47], neurodegeneration diseases [48, 49] and muscular dystrophy [50]. To determine whether UA protects against CuONPs-induced cytotoxicity, we firstly analyzed mitophagy flux in UA-treated EA.hy926 cells. In Mito-DsRed/GFP-LC3 stable cell line, the co-localization of Mito-DsRed (mitochondria) and GFP-LC3 (autophagosomes) revealed that mitophagy was activated in CuONPs- or UA-treated cells. (Fig. 5A). Moreover, UA pretreatment upregulated CuONPs-induced mitophagy level in EA.hy926 (Fig. 5A). FCM and western blotting results showed that UA facilitated removal of damaged mitochondria in CuONPs-treated EA.hy926 cells (Fig. 5C, D). Consistently, UA promoted mtROS clearance in CuONPs-treated cells (Fig. 5E). Then, we investigated the role of PINK1 in UA-treated EA.hy926 cells and the results showed that UA promoted PINK1-GFP recruitment to mitochondria (Fig. 5B). The results also revealed that UA reduced CuONPs-induced cell death in EA.hy926 cells, while *PINK1* knockdown eliminated protective effect of UA against CuONPs-induced cell death (Fig. 5F). These data suggest UA triggers PINK1-dependent mitophagy and protects against CuONPs-induced vascular endothelial cell death.

### Urolithin A alleviates CuONPs-induced vascular injury in mice.

**Fig. 6:**
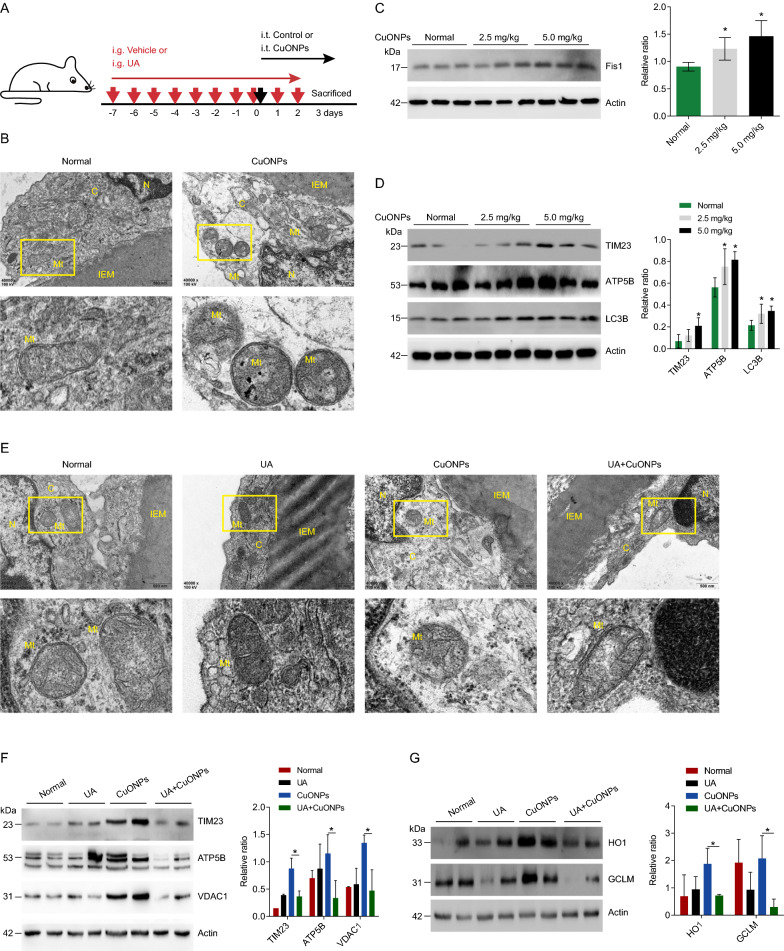
UA alleviated CuONPs-induced vascular injury in mice. **A** Schematic figure for animal experiments. C57BL/6 J mice were treated with vehicle (PBS) or UA (30 mg/kg) for 1 week by intragastric gavage (i.g.), and then exposed to CuONPs via intratracheal instillation (i.t.), meanwhile the mice in UA group and CuONPs + UA group were continuously treated with UA (30 mg/kg) by gavage for 3 days. **B** Representative TEM images of mice abdominal aorta dissected from CuONPs-treated mice. Mice were intratracheally instilled with CuONPs (5 mg/kg) for 3 days. N, nucleus; C, cytoplasm; Mt, mitochondria; IEM, internal elastic membrane. **C** and **D** Western blotting analysis and quantification of protein levels of Fis1, TIM23, ATP5B and LC3B in mice aorta tissues. Mice were intratracheally instilled with 2.5 mg/kg or 5 mg/kg CuONPs for 3 days. Actin was used as the internal control. **E** Representative TEM images of mice abdominal aorta dissected from CuONPs-treated mice. Mice were treated with UA (30 mg/kg) for 10 days by intragastric gavage and then intratracheally instilled with CuONPs (5 mg/kg) at day 7. N, nucleus; C, cytoplasm; Mt, mitochondria; IEM, internal elastic membrane. **F** Western blotting analysis and quantification of protein levels of mitochondria related proteins in aorta tissues dissected from CuONPs-treated mice with or without UA. Actin was used as the internal control. **G** Western blotting analysis and quantification of protein levels of oxidative stress response proteins in aorta tissues dissected from CuONPs-treated mice with or without UA. Actin was used as the internal control. In **C** and **D**, two-tailed unpaired Student’s *t* test was performed for statistical analysis. In **F** and **G**, one-way ANOVA with Tukey’s test was used for multiple comparisons. Results are representative of at least three independent experiments. Data are mean ± S.D. *p < 0.05

To determine the protective role of UA against vascular injury in CuONPs-treated mice, we established CuONPs pulmonary exposure mouse model as described in our previous study [37]. The mice experimental design was illustrated in the Fig. 6A. Through ultrastructural observation under TEM, we showed that CuONPs pulmonary exposure resulted in accumulation of damaged or fragmented mitochondria in mouse aortic endothelial cells (Fig. 6B). Western blotting results revealed that CuONPs increased protein levels of mitochondria fission marker Fis1 and mitochondria related proteins TIM23 and ATP5B in mouse aortic vessels (Fig. 6C, D). Meanwhile, LC3B was upregulated in CuONPs exposure group, indicating CuONPs indeed activated mitophagy flux in mouse aortic vessels (Fig. 6D). Next, we showed that UA obviously alleviated CuONPs-induced mitochondrial dysfunctions (characterized by cristae rarefication and swelling) in vascular endothelial cells (Fig. 6E). Moreover, western blotting results demonstrated that UA significantly decreased TIM23, ATP5B and VDAC1 protein level in aortic vessels of CuONPs-treated mice, indicating that UA accelerated removal of damaged mitochondria in mouse aortic vessels induced by CuONPs exposure (Fig. 6F). Finally, we showed that UA alleviated CuONPs-induced oxidative stress in mouse aortic vessels (Fig. 6G). These data indicate UA induces mitophagy and ameliorates NPs exposure-induced vascular injury.

## Discussions

Mitochondria participate in various aspects of cellular metabolisms, including energy metabolism, biosynthetic hubs, mtROS balance and cellular waste management [51]. Mitochondrial dysfunctions trigger many metabolic diseases, such as cardiomyopathy, respiratory failure, liver failure, diabetes mellitus and neurological disease [27, 52]. It is widely accepted that mitochondrial dysfunctions and mtROS imbalance are main causes of CuONPs-induced cellular toxicity (Table 1). Recently, a study reported that CuONPs was a solid source of oxygen, which directly captured electrons from the respiratory chain and harnessed the trapped electrons for ROS generation. This finding proposed that CuONPs can disturb mitochondrial respiratory, damage cellular energy supply and simultaneously induce mtROS-mediated oxidative stress [53].

**Table 1:** Toxic effects and mechanisms of CuONPs

Nanoparticle source	Particle size	Dose	Exposure time	Cell line/in vivo models	Mechanism of toxicity	References (DOI)
Sigma-Aldrich	< 50 nm	100 mg/kg body weight (intraperitoneal instillation)	14 d	Wistar male rats	Oxidative stress, DNA damage and apoptosis in liver	10.1038/s41598-020-67784-y
Self-synthesized		0, 1.25, 2.5, 5, 10 and 20 μg/ml	48, 72 h	Renal cell carcinoma (786O, A498, CAKI-1, SR786O and HK-2)	ER stress; Apoptosis	10.1016/j.biomaterials.2017.09.008
Sigma-Aldrich	< 50 nm	0–80 μg/ml	4, 24 h	Rat small intestine epithelial cells (IEC-6) 3D human small intestinal tissue model (EpiIntestinal)	ROS production; Cytotoxicity	10.1080/17435390.2019.1578428
Sigma-Aldrich	20–40 nm	10 μg/g body weight (intranasal perfusion)	60 d	Rat liver cells (BRL 3A); Wistar male rats	Oxidative stress, ER stress and apoptosis in liver	10.1016/j.jhazmat.2020.123349
Self-synthesized	141 ± 13 nm in water	0, 10, 100, 1000 μM	5 h	Rat glioma cell line (C6)	ROS production; Cytotoxicity	10.1007/s11064-016-2020-z
Self-synthesized	75 nm	0, 1.25, 2.5 and 5 μg/ml	24 h	Primary human umbilical vein endothelial cells (HUVECs)	Impairment of angiogenesis	10.1039/c3nr04363k
Sigma-Aldrich	< 50 nm	0, 5, 10, 15 and 20 μg/ml	12 h	Murine macrophage J774A.1	Lysosomal damage; Oxidative stress; Inflammation	10.1016/j.jhazmat.2021.125134
Self-synthesized		15.63–625 μM	24 h	Mouse fibroblasts (L929)	DNA damage; ROS production	10.1016/j.tiv.2021.105252
Sigma-Aldrich	< 50 nm	1.25, 2.5 or 5 mg/kg (intratracheal instillation)	3 d	Male C57BL/6 mice	Oxidatives stress; Autophagy dysfunction; Acute lung injury	10.1186/s12951-021-00909-1
Ionic Liquides Technologies	15–100 nm	0–240 μg/ml	18 h	Leukemic cell line (HL60)	Mitochondrial damage; ROS production; DNA damage	10.1016/j.tiv.2015.05.020
Sigma-Aldrich	< 50 nm	0, 10, 20, and 40 μg/ml	0, 1, 3, 6, 12 h	Human umbilical vein endothelial cells (HUVECs)	Lysosomal impairment; ROS production; DNA damage	10.2147/IJN.S241157 10.1016/j.lfs.2020.117571 10.1016/j.freeradbiomed.2018.09.032 10.1016/j.biomaterials.2018.01.048
Sigma-Aldrich	< 50 nm	0, 5, 10, 15, 20 μg/ml (in vitro); 2.5–5.0 mg/kg (intratracheal instillation)	12 h 3 d	Human umbilical vein endothelial cell line (EA.hy926); Male C57BL/6 mice	Mitochondrial dysfunctions; mtROS production; Vascular injury	This study
Sigma-Aldrich		0, 0.01, 0.1, 1, 10, 100 μM	48 h	Human SH-SY5Y neuroblastoma cells (CRL-2266) Human neuroglioma H4 (HTB-148) Rat PC12 (CRL-1721)	ROS production; Apoptosis	10.3390/ijerph17031005
Self-synthesized	124 nm	10 μg/ml	24 h	Human pancreatic cancer cell line (PANC1)	Mitochondrial dysfunctions; ROS production; Apoptosis	10.1038/s41598-019-48959-8
Sigma-Aldrich	< 50 nm	0, 1, 5, 10 and 20 μg/ml	24 h	Human alveolar epithelial cell line (A549) Human cervix carcinoma cell line (HeLa S3)	Cytotoxicity and genotoxicity	10.1186/1743-8977-11-10
Sigma-Aldrich	< 50 nm	30 μg/ml	24 h	Human alveolar epithelial cell line (A549)	Autophagic cell death	10.1371/journal.pone.0043442
Sigma-Aldrich	50 nm	25 μg/ml	24 h	Human alveolar epithelial cell line (A549)	ROS production; Cytotoxicity	10.1021/nn202966t
Self-synthesized			36 h	Human alveolar epithelial cell line (A549)	Inflammation and apoptosis	10.1007/s10529-017-2463-6
Sigma-Aldrich	< 50 nm	0, 2, 4, 6, 8 and 10 μg/ml	24 h	Human colorectal adenocarcinoma cell line (HT29)	Cytotoxicity	10.1007/s00204-017-1976-z
Self-synthesized	30 ± 3.5 nm	0, 4, 6 and 12 μg/ml	24 h	Human breast cancer cells (MCF7)	Apoptosis; Protective autophagy activation	10.1016/j.bbagen.2013.08.011
Sigma-Aldrich	< 50 nm	1, 2, 5, 10, 20, and 40 μg/ml	24 h	Human airway epithelial cells (HEp-2)	Oxidative damage; ROS generation; Apoptosis	10.1007/s12011-022-03107-8
Nanostructured & Amorphous Materials Inc	50 nm	2.5–5.0 mg/kg (intratracheal instillation)	0, 1, 3, 7 d	Female C57BL/6 mice	Protein chlorination and acute lung inflammation	10.3390/ijms22179477
Sigma-Aldrich	< 50 nm	0, 25, 50, and 100 μg/kg (intratracheal instillation)	48 h	Female BALB/c mice Human airway epithelial cell line (NCI-H292)	Inflammatory responses and collagen deposition in lung tissue	10.1080/17435390.2018.1432778
Sigma-Aldrich	46.5 nm	1, 2.5, 5, and 10 mg/kg body weight (nasal instillation)	7, 14 and 28 d	C57BL/6 mice Alveolar and bronchial epithelial cell lines (A549 and BEAS-2B)	Pulmonary infammation and fibrosis in mice Cytotoxicity and apoptosis in epithelial cells	10.1038/s41598-018-22556-7
Self-synthesized	40 nm to 110 nm	0, 0.625, 1.25, 2.5 and 5 μg/ml	48 h	Bladder cancer cell lines (T24, J82, 5637, and UMUC3)	ROS production; Cell cycle; Apoptosis	10.1038/cddis.2013.314
Intrinsiq Materials Ltd	28.2 ± 13.7 nm	10 μg/ml	0, 1, 3, 6, 12, 24, 48 h	Alveolar epithelial cell line (A549)	Oxidative stress; Apoptosis	10.1186/s12989-016-0160-6
Self-synthesized	4, 24 nm	0, 0.5, 1.0, 1.5 and 2.0 mM	0, 1, 4, 24, 48 h	Alveolar epithelial cell line (A549)	ROS production; Cytotoxicity	10.1039/c5en00271k
Sigma-Aldrich	20–40 nm	0, 1, 5 and 10 μg/ml	4 h	Alveolar and bronchial epithelial cell lines (A549 and BEAS-2B)	DNA damage; Cytotoxicity	10.1002/smll.201201069

Mammalian cells have evolved several mechanisms for maintaining mitochondrial homeostasis, such as mitochondrial fission–fusion dynamics [54]. Mitochondrial fission is involved in mitochondria biogenesis and mitochondria quality control by promoting elimination of damaged mitochondria. Mitochondrial fusion participates in cellular stress response through rescuing non-functional mitochondria [38, 46, 55]. Mitochondrial dynamics imbalance was involved in multiple NPs-induced toxicity [32, 33, 56]. In this study, we demonstrated that CuONPs exposure caused oxidative stress and consequently induced mitochondria fission in vascular endothelial cells characterized by increases of fission mediators Fis1 and p-Drp1 (Fig. 1A, B, D, E). It is well documented that excessive mtROS-mediated mitochondrial stress contributes to mitochondria fission [54]. Mitochondrial superoxide anions (O_2_^•−^) is the major form of mtROS. We previously reported that CuONPs exposure resulted in accumulation of mitochondrial O_2_^•−^ in vascular endothelial cells [28, 29]. Consequently, excessive mitochondrial O_2_^•−^ triggered oxidative DNA damage and p38 MAPK-dependent vascular endothelial cells death [57]. In this study, we further revealed that mitochondrial O_2_^•−^ was involved in CuONPs-induced mitochondrial fission in that ROS scavenger NAC decreased p-DRP1 and Fis1 and significantly inhibited CuONPs-induced mitochondrial fission (Fig. 1B, E). Consistently, mitochondrial O_2_^•−^ promotes mitochondrial fission in coronary endothelial cells, while O_2_^•−^ scavenger TEMPOL significantly inhibits mitochondrial fission in Diabetic mice [58]. In ischemia/reperfusion (I/R) cell model, I/R-induced mitochondrial fission is also obviously inhibited by O_2_^•−^ scavenger [59]. All these results indicate mitochondrial O_2_^•−^ functions as an up-stream signal of mitochondrial fission.

Currently, mitochondrial fission contributes to pro-survival or pro-death in cells under oxidative stress is still controversial. An early study demonstrated that inhibition of mitochondrial fission prevented high glucose-induced cardiomyocytes apoptosis [60]. Clare Sheridan et al. reported that mitochondrial fission induced by pro-apoptotic BCL2-associated X protein (BAX) and the release of pro-death cytochrome *c* were two separable events [61]. In contrast with these findings, a recent study reported that anti-apoptotic protein Myeloid cell leukemia 1 (MCL-1) induced mitochondrial fission and inhibit cardiomyocytes death, suggesting that mitochondrial fission protects against stress-induced cell death [62]. Masahiro Morita et al. recently found that nutrient-sensing regulator mTOR complex 1 (mTORC1) regulated mitochondrial dynamics, and mTOR inhibitor prevented mitochondrial fission and consequently triggered apoptosis [63]. Accordingly, mitochondrial fission may be bad or good and therefore plays dual roles in different physiological context. In this study, we found that mitochondrial fission inhibitor Mdivi-1 aggravated CuONPs-induced oxidative stress and cells death in EA.hy926 (Fig. 1F, G). This indicates that mitochondrial fission is a crucial pro-survival process in CuONPs-induced cytotoxicity.

Accumulating evidence has highlighted that mitochondrial fission contributes to removal of damaged mitochondria via autophagy (a process termed as mitophagy) [64–66], through which protects against many types of NPs-induced cytotoxicity [34–36, 67]. Mitophagy is regulated by ubiquitin kinase PTEN induced kinase 1 (PINK1) and mitophagy receptors including Tax1 binding protein 1 (TAX1BP1), Calcium binding and coiled-coil domain 2 (CALCOCO2, NDP52), Optineurin (OPTN), Neighbor of BRCA1 gene 1 protein (NBR1) or Sequestosome-1 (SQSTM1/p62) [44]. After mitochondria dysfunction, excessive mtROS reduces mitochondrial membrane potential and induces PINK1 stabilization on the outer mitochondrial membrane (OMM) of damaged mitochondria, where it phosphorylates the ubiquitin of OMM proteins and then recruits mitophagy receptors to trigger mitophagy [44]. Defective mitophagy leads to accumulation of unwanted or damaged mitochondria, which results in excessive mtROS production and cell death via mitochondrial-dependent apoptosis pathway. Thus, clearance of damaged mitochondria via mitophagy is a potential therapeutic intervention to multiple diseases such as cancers, Alzheimer’s disease and sepsis-induced heart failure [68–70]. Therefore, we hypothesized that the interplay between CuONPs-triggered mitochondrial fission and mitophagy might be involved in CuONPs-induced cytotoxicity. We showed that CuONPs exposure resulted in autophagosome recruitment to damaged mitochondria in vascular endothelial cells (Fig. 2A). Meanwhile, CuONPs upregulated PINK1 level in CuONPs-treated EA.hy926 cells (Fig. 3A). In addition, we found that PINK1 was recruited to damaged mitochondria (Fig. 3B) and *PINK1* knockdown inhibited clearance of damaged mitochondria and mtROS, consequently aggravated cell death in CuONPs-treated EA.hy926 cells (Fig. 3D–F). These data indicate that PINK1-dependent mitophagy facilitates removal of damaged mitochondria to appropriately mitigate oxidative stress and cell death in CuONPs-treated EA.hy926 cells. Recently, we reported that CuONPs exposure increased intracellular copper ions concentration both in vitro and in vivo model [16, 37]. Copper is an essential metal regulator of autophagy initial kinases ULK1/2, which directly interacts with ULK1/2 and enhances these kinases activity, consequently promotes autophagic flux [71]. Moreover, copper ions also upregulate PINK1 in primary chicken hepatocytes [72]. These findings indicate copper ions promotes PINK1-dependent autophagy/mitophagy in CuONPs-treated cells. However, the interactions among copper ions, mitochondrial fission and mitophagy need further investigations.

Subsequently, we identified that TAX1BP1 was the primary adaptor linking autophagosomes and ubiquitinated mitochondria via siRNA screening. Compared to other mitophagy receptor genes, *TAX1BP1* knockdown significantly aggravated CuONPs-induced cell death in EA.hy926 (Fig. 4A). Furthermore, TAX1BP1 was recruited to autophagosomes and mediated the connecting between damaged mitochondria and autophagosomes in CuONPs-treated EA.hy926 cells (Fig. 4B). *TAX1BP1* knockdown obviously caused more accumulation of damaged mitochondria and mtROS in CuONPs-treated EA.hy926 cells (Fig. 4C, D). Shireen A. Sarraf et al. showed that TAX1BP1 participated in clearance of stress-induced protein aggregates [73]. TAX1BP1 also promotes autophagy and contributes to metabolic transition of activated immune T Cells [73]. These data indicate TAX1BP1 serves multiple functions in different cells or diseases model. Notably, NDP52 and OPTN, besides TAX1BP1, are the major receptors mediating PINK1-mediated mitophagy in human cervical cancer cell line (HeLa) [44]. In the current study, we showed that either *NDP52* or *OPTN* knockdown aggravated CuONPs-induced cell death, but their protective effects is less than *TAX1BP1* (Fig. 4A), suggesting the unique characteristics of mitophagy profile in CuONPs-treated vascular endothelial cells.

Considering mitophagy is a primary process facilitating clearance of damaged mitochondria and excessive mtROS induced by CuONPs, the means for the restoration of appropriate mitophagy is a promising strategy to protect against NPs exposure-induced toxicity. Urolithin A (UA) is a natural gut microbiome-derived metabolite. UA was reported to protect against oxidized low-density lipoprotein (ox-LDL)-induced vascular endothelial dysfunction by upregulating levels of nitric oxide and endothelial nitric oxide synthase, meanwhile markedly reducing the expression of inflammatory cytokines ICAM-1 and MCP-1 [74]. Several exciting studies have showed that UA is a first-in-class natural compound that inducing autophagy/mitophagy. It prolonged lifespan in *Caenorhabditis elegans* and improved muscle functions in mouse model by restoring mitochondrial respiratory capacity and removal of dysfunctional mitochondria [47, 50, 75]. Lee et al. also reported that UA inhibited mitochondrial calcium influx and mtROS accumulation in neuronal cells induced by high glucose, and consequently prevented diabetes mellitus-associated neurodegenerative disease pathogenesis [49]. Wang et al. showed that UA-induced mitophagy modulated mitochondrial unfolded protein response to attenuate sepsis-triggered myocardial stress [76]. These findings indicate UA is a promising therapy candidate for preventing NPs exposure-induced toxicity by maintaining mitochondrial and mtROS homeostasis. In this study, we demonstrated that UA promoted removal of damaged mitochondria and accelerated elimination of excessive mtROS in CuONPs-treated cells via PINK1-dependent pathway (Fig. 5). Furthermore, we revealed that UA significantly reduced damaged mitochondria mass and alleviated vascular injury in CuONPs-treated mice (Fig. 6D). These findings suggest mitophagy effectively alleviates CuONPs vascular toxicity. In addition, previous studies demonstrated that UA regulated mitochondrial functions by maintaining mitochondrial calcium homeostasis, regulating cellular NAD + metabolism and activating mitochondrial unfolded protein response [49, 76, 77]. Thus, a combination of restoring mitochondrial functions and triggering mitophagy-mediated removal of damaged mitochondria is a promising strategy to alleviate NPs pulmonary exposure-induced vascular injury and related cardiovascular diseases.

## Material and Methods

### Materials and reagent

CuONPs (#544868), mitochondrial division inhibitor 1 (Mdivi-1, #M0199) and chloroquine (CQ, # C6628) were purchased from Sigma Aldrich (St. Louis, MO, USA). Wortmannin (Wort, #S2758) was purchased from Selleck Chemicals (Shanghai, China). Urolithin A (UA, #HY-100599) were purchased from MedChemExpress (Shanghai, China). MitoTracker™ Green FM(#M7514), MitoSOX™ Red (#M36008) and Dulbecco’s modified Eagle’s medium (DMEM, #11965-092) were purchased from Thermo Fisher Scientific (Waltham, MA, USA). Fetal bovine serum (FBS, #04-001-1ACS) was purchased from Biological Industries (Kibbutz Beit Haemek, Israel).

### Cell culture and treatment

The EA.hy926 cell line (established by fusing primary human umbilical vein cells with a thioguanine-resistant human lung carcinoma cell line A549) and HEK293T/17 lentiviral packaging cell line were purchased from the National Collection of Authenticated Cell Cultures (Shanghai, China). All cells were cultured in DMEM medium supplemented with 10% FBS and 100 U penicillin–streptomycin at 37 °C with 5% CO_2_. For CuONPs treatment, EA.hy926 cells were seeded in 12-well plates for overnight and then treated with different concentration of CuONPs for indicated time points. CuONPs was diluted in culture medium and vortexed for several seconds before treatment. For treatment with chemical materials, cells were pretreated with selected chemicals 1 h before CuONPs treatment.

### Generation of stable cell lines

The plasmids mCherry-Parkin (Plasmid #23956), GFP-LC3 (Plasmid #11546), psPAX2 (Plasmid #12260) and pMD2.G (Plasmid #12259) were obtained from Addgene (Cambridge, MA, USA). The plasmid pDsRed2-Mito was purchased from Clontech (Mountain View, CA, USA). The cDNA cloning and expression plasmid pCDH-CMV-MCSEF1-Puro was obtained from System Biosciences (Palo Alto, CA, USA). For lentiviral vectors construction, gene fragments were PCR amplified using I-5™ Hi-Fi DNA Polymerase (#PDP-100, MCLAB, San Francisco, CA, USA) and then inserted into pCDH-CMV-MCSEF1-Puro lentivirus vector using pEASY®-Basic Seamless Cloning and Assembly Kit (#CU201-02, TransGen Biotech, Beijing, China). Then, the sequenced lentivirus plasmid was co-transfected with packaging plasmids psPAX2 and pMD2.G into HEK293T/17 using NEOFECT™ DNA transfection reagent (Neofect biotech, Beijing, China) for 48–72 h. The culture supernatants were harvested and inoculated into EA.hy926 cells. The stable cell lines were generated by two rounds of puromycin selection.

### Animals and treatment

The physical–chemical characteristics of CuONPs were described in our previous study [37]. For preparation of CuONPs suspension, CuONPs were firstly diluted in serum solution (composed of 2% heat-inactivated sibling mouse serum in MilliQ water) to the concentration of 2 mg/mL. Then CuONPs solution was sonicated for 20 min with an ultrasonic cleaner. CuONPs suspension was freshly prepared before intratracheal instillation to avoid dissolution of CuONPs to copper ions. Before instillation, CuONPs suspension was thoroughly vortexed for 30 s to avoid any agglomeration.

This study was approved by the Institutional Animal Care and Use Committee of Chongqing Medical University. Specific-pathogen-free C57BL/6 J male mice (aged 8–12 weeks, weight 18–22 g) were purchased from the Experimental Animal Center of Chongqing Medical University. All animals were randomized into the following 4 groups: Control, CuONPs (5 mg/kg), UA (30 mg/kg), and CuONPs (5 mg/kg) + UA (30 mg/kg). The animals were housed in groups in controlled environmental temperature. After 1-week of habituation, the mice in UA group and CuONPs + UA group were treated daily orally with UA (30 mg/kg) by gavage for 7 days. On the eighth day, the mice in CuONPs group and CuONPs + UA group were intratracheally instilled CuONPs after anesthetization with 1% pentobarbital sodium, meanwhile the mice in UA group and CuONPs + UA group were continuously treated with UA (30 mg/kg) by gavage for 3 days. Finally, the C57BL/6 J mice were sacrificed and the abdominal aorta were obtained.

### Transmission electron microscopy (TEM) imaging

After CuONPs treatment, cells were centrifuged at 106 × g for 5 min after trypsinization and then fixed with 4% glutaraldehyde for 2 h at 4 °C. Next, cells were postfixed with 1% osmium tetroxide for 1 h at 4 °C. Subsequently, cells were dehydrated in a grade series of alcohol and acetone followed by embedment in Epon 816 (Electron Microscopy Sciences, Hatfield, PA, USA). Ultrathin sections were obtained by using a Leica EM UC7 Ultramicrotome (Leica Microsystems, Buffalo Grove, IL, USA) and stained with uranyl acetate and lead citrate. TEM images were taken with a JEM-1400 Plus transmission electron microscope (JEOL Ltd. Tokyo, Japan).

### Western blotting

The cells seeded in 12-well plate were washed with cold phosphate-buffered saline (PBS) buffer and lysed with 2 × protein lysis buffer (2% SDS, 5% 2-mercaptoethanol, 0.5% sucrose and 0.2% bromophenol blue). Mouse vascular tissues were washed with cold PBS and lysed with RIPA buffer containing protease inhibitors (Beyotime, Shanghai, China, #P0013B). Then, lysed tissues were homogenized with dounce homogenizer and centrifuged at 10,000 × g for 10 min at 4 °C. Protein lysates were boiled for 5 min and separated in 8%-12% polyacrylamide gels. After electrophoresis, proteins were transferred from gel to polyvinylidene fluoride membrane (Merck Millipore, Billerica, MA, USA). The membrane was incubated with 5% non-fat milk, incubated separately with primary antibodies diluted in blocking buffer for overnight at 4 °C and then probed with secondary antibody diluted in TBST (Tris Buffered Saline with Tween 20) at room temperature for 1 h. After washing with TBST, the membranes were incubated with BeyoECL Star substrates (Beyotime, #P0018AM). All antibodies used in this study were listed as below: LC3B (1:3,000, Sigma Aldrich, #L7543), ATP5B (1:500, Santa Cruz Biotechnology, #sc-166462), TOM20 (1:1,000, Proteintech, Wuhan, China, #11802-1-AP), TIM23 (1:1,000, Santa Cruz Biotechnology, #sc-514463), VDAC1(1:2,000, Bimake, #A5224), FIS1 (1:3,000, Proteintech, #10956-1-AP), DRP1 (1:4,000, Proteintech,#12957-1-AP), p-DRP1 (1:1,000, Cell Signaling Technology, #3455S), GAPDH (1:10,000, Proteintech, #60004-1-lg), Actin (1:10,000, TransGen Biotech, Beijing, China, #HC201-01), HRP-conjugated goat anti-mouse IgG (1:10,000, TransGen Biotech, #HS201-01) and HRP-conjugated goat antirabbit IgG (1:10,000, TransGen Biotech, #HS101-01).

### Flow cytometry

After treatment, cells seeded in 12-well plate were washed with PBS and incubated with 0.25% trypsin/EDTA solution (Beyotime, #C0201). Then, cells were pipetted and collected through centrifugation, and incubated with MitoTracker™ Green FM (500 nM, Thermo fisher, #M7514) or MitoSOX™ Red (5 μM, Thermo fisher, #M36008) for 15–30 min at 37 °C in a cell culture incubator. The stained cells were re-pelleted and then detected under a CytoFLEX flow cytometry (Beckman Coulter, Miami, FL, USA). The cytometry data were analyzed by FlowJo™ v10 Software BD Biosciences (San Jose, CA, USA).

### Immunofluorescence cell staining

The cells were seeded on a sterilized coverslip in a 24-well plate for overnight. After treatment, cells were fixed in 4% paraformaldehyde for 15 min at room temperature, permeabilized with 0.2% Triton X-100 for 10 min at room temperature, and then blocked with blocking buffer (2% BSA and 0.3 M glycine in PBS) for 1 h at room temperature. The cells were then incubated with primary antibodies diluted in blocking buffer for overnight at 4 °C. After washing three times with PBS, cells were incubated with Alexa Fluor-conjugated secondary antibodies and 4′,6-diamidino-2-phenylindole (DAPI, Molecular Probes, Eugene, OR, USA) diluted in blocking buffer for 1 h at room temperature. After washing with PBS, cells were sealed with nail polish. Fluorescence images were taken under a Nikon A1R + /A1 Confocal Microscope (Nikon, Tokyo, Japan). The antibodies used in this study are as follows: ATP5B (1:25, Santa Cruz Biotechnology, #sc-166462), TAX1BP1(1:50, Proteintech, #14424-1-AP), Alexa Fluor 594-conjugated donkey anti-rabbit IgG (1:500, Molecular Probes, #A-21207) and Alexa Fluor 647-conjugated donkey anti-rabbit IgG (1:500, Molecular Probes, #A-21245).

### Cell viability assays

The cells were cultured in 96-well plates at a density of 1 × 10^4^/well for overnight. After treatment for indicated time points and concentrations described in figure captions, CellTiter 96® AQueous One Solution Reagent (#G3581, Promega, Madison, WI, USA) was diluted in culture media and added to each well of culture plates. The plate was incubated at 37 °C for 1 h and the absorbance of each well was measured in a VERS Amax Microplate Reader (Molecular Devices, Sunnyvale, CA, USA) set at 490 nm and 630 nm. All assays were conducted in triplicate and repeated three times.

### Transfecting siRNA using lipofectamine™ RNAiMAX

Before siRNA duplex transfection, cells seeded in 12-well plates for overnight were added fresh culture media. The siRNA duplex and Lipofectamine RNAiMAX transfection reagent (Invitrogen, Waltham, MA, USA) were diluted in Opti-MEM media (GIBCO, Grand Island, NY, USA), respectively. Then, the diluted siRNA duplex and Lipofectamine RNAiMAX were mixed gently and incubated for 10–20 min at room temperature. Finally, the RNAi duplex-Lipofectamine™ RNAiMAX complexes were added into plate well. After siRNA transfection for 36–48 h, RNAi efficiency was analyzed by real-time quantitative PCR or western blotting. All siRNA duplex used in this study were synthesized by GenePharma (Shanghai, China) and the target sequences were listed in Table 2.

**Table 2:** The list of siRNA sequences used in this study

Target	Sequence
PINK1 (human)	GAGAAGUGUUGUGUGGAAATT
p62/SQSTM1(human)	GCAUUGAAGUUGAUAUCGATT
NDP52 (human)	GCUUGUUCAGGGAGAUCAATT
NBR1 (human)	GCUCAAAGAUGAAGUUCAATT
TAX1BP1 (human)	CAAAGAAAUUGCUGACAAATT
Optineurin (human)	GGAAGUUUACUGUUCUGAUTT
GFP (human)	GCAGCACGACUUCUUCAAGTT

### Real-time quantitative PCR (qPCR)

Total RNA was extracted from cells using Eastep Super Total RNA Extraction Kit (# RC112-01, Vazyme, Nanjing, China) and then the purified RNA was reversely transcribed to cDNA with HiScript II Q RT SuperMix for qPCR (+ gDNA wiper) (#R223-01, Vazyme) according to the manufacturer protocols. qPCR amplification assays were performed with ChamQ Universal SYBR qPCR Master Mix (#Q711-03, Vazyme) under a CFX Connect Real-Time PCR Detection System (Bio-Rad, CA, USA). The *TBP* (TATA-Box Binding Protein) was chosen as housekeeping reference gene. The specific primers were synthesized by Sangon (Shanghai, China) and shown in Table 3**.**

**Table 3:** The list of QPCR primers used in this study

Gene names	Forward primer(5´-3´)	Reverse primer(5´-3´)
*NDP52*	CTGCTATGTGGATGAGGAT	ATGTCTGAGTTCTGCTTCT
*NBR1*	ACAGCATAAGTGACATCCT	TTCTGGTATGGTAACTGGTAA
*TAX1BP1*	GCCAAGAGTTACCTTCCTA	TTGACTGTTGATCCTTCCA
*OPTN*	GCTCCTCAGAAGATTCCTT	TGCTCCTATATTTAGACAATGC
*p62*	TCGGATAACTGTTCAGGAG	CGGATTCTGGCATCTGTA
*PINK1*	GCCATCTTGAACACAATGA	CTGTAAGTGACTGCTCCA
*TBP*	ATCAGTGCCGTGGTTCGT	TTCGGAGAGTTCTGGGATTG

### Statistical analysis

The data were shown as mean ± standard deviation (S.D.). Each experiment was repeated three times. Data were analyzed by two-tailed unpaired Student's *t*-test for two groups comparisons or one-way ANOVA followed by Tukey's test for comparisons of parameters among multiple groups. All statistical analyses were taken with GraphPad Prism 5.0 software (San Diego, CA, USA). *p < 0.05 was considered statistical significance.

## Data Availability

The authors declare that data related to this study are provided upon request.
